# Self-Assembly Iron Oxide Nanoclusters for Photothermal-Mediated Synergistic Chemo/Chemodynamic Therapy

**DOI:** 10.1155/2021/9958239

**Published:** 2021-04-07

**Authors:** Xiang Li, Zhen Wang, Mian Ma, Zhouqing Chen, Xiang-long Tang, Zhong Wang

**Affiliations:** ^1^Department of Neurosurgery & Brain and Nerve Research Laboratory, The First Affiliated Hospital of Soochow University, Suzhou, Jiangsu Province, China; ^2^Department of Neurosurgery, The Affiliated Hospital of Xuzhou Medical University, Xuzhou, Jiangsu Province, China; ^3^Department of Neurosurgery, The Affiliated Brain Hospital with Nanjing Medical University, Fourth Clinical College of Nanjing Medical University, Jiangsu Province, China; ^4^Department of Neurosurgery, Suzhou Municipal Hospital, Suzhou, Jiangsu Province, China

## Abstract

**Methods:**

Superparamagnetic iron oxide nanoclusters (SPIOCs) were located within the core, which resulted in high photothermal conversion and outstanding generation of reactive oxygen species (ROS). The shell consisted of a human serum albumin- (HSA-) paclitaxel (PTX) layer, which extended the blood circulation time and ensured the effectiveness of the chemotherapy. Arg-Gly-Asp peptides (RGD) were linked to the naked cysteine moieties in HSA to promote the specific targeting of human glioma U87 cells by *α*_v_*β*_3_ integrins. Continuous near-infrared light irradiation triggered and promoted the synergistic chemo/CDT therapy through the photothermal effect.

**Results:**

Our SPIOCs@HSA-RGD nanoplatform showed well biocompatibility and could target glioma specifically. Photothermal conversion and ROS burst were detected after continuous 808 nm light irradiation, and a significant antitumor effect was achieved.

**Conclusion:**

Experimental in vitro and in vivo evaluations showed that our photothermal-mediated chemo/CDT therapy could efficiently inhibit tumor growth and is therefore promising for cancer therapy.

## 1. Introduction

In recent decades, conventional therapies for glioma have mainly comprised surgery, chemotherapy, and radiotherapy; however, their therapeutic effect has not been satisfying [1, 2]. Traditional chemotherapy suffers from poor specificity and multiple drug resistance (MDR) and shows substantial toxicity to normal tissues [3–6]. Specific targeting capacity to tumor sites and effective antitumor outcomes are urgently required. Nowadays, nanotechnology-based therapies have presented many promising advantages in this area [7]. Considering the leakage of blood-brain barrier (BBB) and the enhanced permeability and retention (EPR) effect in glioma [8, 9], nanoagents offer unparalleled advantages of penetration and retention in tumor tissue [10], which is of great significance to glioma treatment.

Among various nanocarriers, iron oxide nanoparticles (Fe_3_O_4_ NPs) show superior properties, such as low toxicity and excellent biocompatibility and biodegradability, as well as superior photothermal effects [11–14]. Ferumoxytol, an iron oxide-based MRI T_2_ contrast agent approved by the U.S. Food and Drug Administration (FDA), has been reported [15]. It has been found that some characteristics of Fe_3_O_4_ NPs are enhanced in ultrasmall superparamagnetic iron oxide nanoclusters (SPIOCs) [16–18]. Once undergoing endocytosis by cancer cells, Fe_3_O_4_ NPs could function as an effective catalyst of Fenton reaction to induce cancer cell death. In addition, the properties of easy preparation, low cost, and controllable size increase the prospects for clinical applications [19]. Meanwhile, under consideration of naked Fe_3_O_4_ NPs which are not fully biocompatible, human serum albumin (HSA), with its inherent biocompatibility and abundance, is usually used to decrease their toxicity [20]. These HSA-coated nanoparticles can be internalized into cancer cells by gp60 receptor-related translation mechanism that overcomes negative charge-induced repulsion. In addition, Arg-Gly-Asp (RGD) peptide, a classical binding motif targeting *α*_v_*β*_3_ integrins of tumors, could enhance the cancer binding affinity and cellular uptake of nanoparticles [21]. Thus, a nanosystem could specifically accumulate in glioma tissues by enhanced permeable reaction (EPR) as well as the assistance of RGD peptide [22].

Photothermal therapy is a noninvasive and controllable strategy superior to other traditional therapies [23]. Since any single therapy still suffers from limited efficacy, photothermal combination therapies are used to increase the efficacy of chemotherapeutic drugs for destroying tumor tissues at the same dose, and they are commonly combined with other therapies, such as chemotherapy and chemodynamic therapy (CDT) [24, 25]. Although reactive oxygen species (ROS) mediate physiological and pathophysiological signal transduction, excessive cytotoxic ROS, which cause oxidative stress, lipid peroxidation, and DNA damage in tumors [26, 27], have recently been used to treat glioma [28]. Exogenous ROS, such as hydroxyl radicals (^·^OH) and superoxide anions (O_2_^−^) are toxic and have high oxidation capability [29]. In the presence of iron oxide NPs, large amounts of ROS are produced by the Fenton reaction, which converts hydrogen peroxide (H_2_O_2_) into cytotoxic hydroxyl radicals (^·^OH); this reaction is promoted by the higher local temperature generated by the photothermal effect [30–32]. Excessive ROS could not only promote chemotherapy but also stimulate drug release from nanocarriers, which is of great significance in tumor chemotherapy [33, 34].

**Figure 1:**
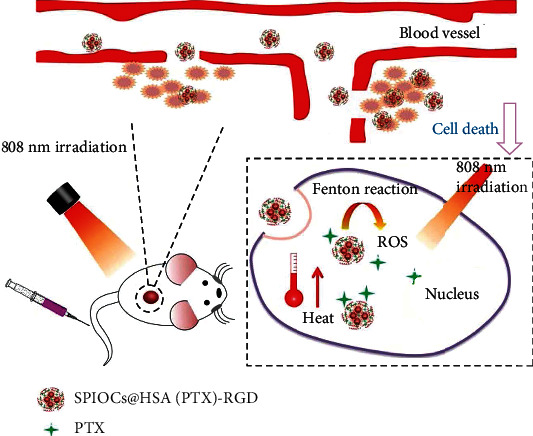
Schematic illustration of SPIOCs@HSA(PTX)-RGD nanoplatforms applied in photothermal mediated chemo/chemodynamic therapy.

In our work, we aimed to promote the synergy efficiency of chemotherapy and chemodynamic therapy by means of ROS burst from the Fenton reaction mediated by photothermal conversion (Figure 1). We designed a novel nanoplatform (SPIOCs@HSA(PTX)-RGD) with superparamagnetic Fe_3_O_4_ nanoclusters (SPIOCs) as the spheroid core coated with a HSA-PTX layer. The SPIOCs@HSA(PTX)-RGD nanoplatform showed good biocompatibility and excellent specific targeting, which was attributed to the HSA and RGD ligands. It could be internalized in tumor cells via endocytosis and accumulated in sufficient concentrations through enhanced permeable reaction (EPR). Irradiation with near-infrared (NIR) laser light (808 nm) resulted in enhanced release of drugs. Further, the catalytic SPIOCs and SPIOC-induced photothermal conversion promoted the iron-mediated Fenton reaction to generate hydroxyl radicals and enhance the ROS level, which triggered tumor apoptosis and ablation. Photothermal-mediated chemo/chemodynamic therapy could inhibit tumor growth more efficiently than any single therapy. The antitumor efficiency of the SPIOCs@HSA(PTX)-RGD nanoplatform was demonstrated by both in vivo and in vitro experiments.

## 2. Material and Methods

### 2.1. Materials

Iron(III) acetylacetonate (Fe(acac)_3_), oleic acid, oleylamine, and ethanol were purchased from Aladdin (China). DMEM, fetal bovine serum (FBS), and penicillin-streptomycin were purchased from Gibco (USA). HSA was purchased from Beijing Biosynthesis Biotechnology Co., Ltd. Thiolated RGD peptides were obtained from China Peptides Co., Ltd. Sulfo-SMCC, 4,6-diamidino-2-phenylindole (DAPI), and DCFH-DA probe were purchased from Sigma-Aldrich (USA). TUNEL apoptosis assay kit was obtained from Beyotime Biotechnology (China).

### 2.2. Preparation of Ultrasmall Superparamagnetic Iron Oxide Nanoparticles

Ultrasmall superparamagnetic iron oxide nanoparticles were synthesized by the procedure reported in a previous study [35]. In brief, equal volumes of oleic acid and oleylamine (15 mL), Fe(acac)_3_ (9 mmol) and benzyl ether (40 mL) were mixed and homogenized by mild mechanical stirring. The temperature was continuously heated to 210°C, and the solution was refluxed at this temperature for 6 hours under a nitrogen atmosphere. Superparamagnetic iron oxide NPs were synthesized when the solution color changed from brown to black and were precipitated with excess ethanol. Then, superparamagnetic iron oxide NPs were separated by centrifugation. After washing with absolute ethanol, the particles were resuspended in chloroform.

### 2.3. Synthesis of SPIOCs@HSA(PTX) NPs

SPIOCs@HSA(PTX) NPs were synthesized by the following steps: monodispersed iron oxide nanoparticles were dissolved in chloroform and then mixed with HSA solution (pH 7.4, 26.5 mg/mL). Ultrasound vibration was carried out at 5 min intervals to prevent the generation of high temperatures. After evaporation of the organic chloroform phase by a rotary evaporator, a diaphanous SPIOCs@HSA solution was obtained. PTX was dissolved in DMSO and added dropwise to the SPIOCs@HSA solution under ultrasonic vibration for at least 5 min. SPIOCs@HSA(PTX) NPs were obtained after dialysis with a membrane (MW: 8 kDa).

### 2.4. Synthesis of SPIOCs@HSA(PTX)-RGD NPs

Thiolated RGD peptide was conjugated to enhance the tumor-targeting specificity with a method similar to that described in our previous report [36]. In brief, SPIOCs@HSA(PTX) solution (pH 7.4) was incubated with Sulfo-SMCC dissolved in DMSO. The solution was placed in an oscillating shaker (130 rpm) for half an hour at 25°C. Subsequently, maleimide-decorated SPIOCs@HSA(PTX)-Mal were centrifuged (16000 g), and thiolated RGD peptide was added to the SPIOCs@HSA(PTX)-Mal solution (pH 7.4) at 4°C overnight. After centrifugation in filtration tubes (MWCO = 8000-12000 Da), purified SPIOCs@HSA(PTX)-RGD NPs were obtained.

### 2.5. Characterization of the Nanoparticles

The morphology was characterized by transmission electron microscopy (TEM, JEOL JEM-2100). The hydrodynamic radii and zeta potentials of the nanoparticles were measured by a laser granularity analyzer (Malvern Zetasizer, UK).

### 2.6. Photothermal Conversion Efficiency In Vitro

An aqueous solution of SPIOCs@HSA(PTX)-RGD NPs (60 *μ*g Fe/mL) was irradiated by 808 nm near-infrared laser (0.5 W/cm^2^) to evaluate the photothermal properties. An infrared thermal imager (Fluke TiS20) equipped with a temperature-sensitive probe was used to monitor the real-time thermal behaviors per second during irradiation.

### 2.7. Cellular Uptake

Human glioma U87 cells in logarithmic growth were seeded in confocal laser petri dishes (diameter: 35 mm) at the density of 10^5^/well and cultured in DMEM with 10% FBS for 24 hours (saturated humidity, 5% carbon dioxide, 37°C). Then, the U87 cells were incubated with Ce6-labeled SPIOCs@HSA-RGD NPs for 4 hours. Afterwards, they were fixed with 4% paraformaldehyde, and their nuclei were labeled with DAPI. The U87 cells were washed with PBS solution (pH 7.4) several times, and the fluorescence signals were observed by confocal laser scanning microscopy (CLSM).

### 2.8. Cell Viability Assays

The cell cytotoxicity was measured by the MTT assay. Human glioma U87 cells were seeded and cultured in 96-well plates (DMEM medium with 10% FBS and 1% penicillin-streptomycin). The SPIOCs@HSA(PTX), SPIOCs@HSA(PTX)-RGD, and SPIOCs@HSA(PTX)-RGD+irradiated groups with different PTX concentrations were incubated with the U87 cells, and PBS was used as the control. After 12 h of incubation, the SPIOCs@HSA(PTX)-RGD+irradiated group was exposed to 808 nm NIR laser illumination (0.5 W/cm^2^) for half an hour after the culture medium was replaced by PBS. Then, the cells were incubated with fresh culture medium for 24 h. Finally, the MTT assay was carried out. To prevent drugs from reacting with MTT, the original culture medium was discarded, and MTT-containing culture medium (serum-free medium : MTT = 5 : 1) was added. After 4 h of culture in the dark, the medium was discarded, and DMSO was added. All wells were mixed evenly by placing the plate on a shaker, and the absorbance was measured with a microplate reader.

### 2.9. Animal Model

Male Balb/c nude rats (4-5 weeks) were purchased from Nanjing Biomedical Research Institute of Nanjing University, and subcutaneous xenograft models were established; all experiments were carried out under the permission and guidelines of the Ethics Committee of Nanjing Medical University. The U87 cells were collected by resuspension and centrifugation in serum-free medium, and 3 × 10^6^ cells were injected into the right flank region of each rat subcutaneously. The rats were housed in an SPF-grade environment.

### 2.10. In Vivo Fluorescence Imaging and Biodistribution

Two hundred microliters of HSA(PTX), SPIOCs@HSA(PTX), and SPIOCs@HSA(PTX)-RGD was injected into randomly selected tumor-bearing nude rats via the tail vein (labeled with Ce6, Ce6: 1.90 mg/kg). After administration of anesthesia with isoflurane, in vivo biodistributions were detected by IVIS spectrum small-animal imaging system (PerkinElmer, USA). The rats were euthanized under thoroughly anesthetizing with isofluorane 10-hour postinjection. Tumors and normal organs (heart liver, lungs, kidneys, and spleen) were excised immediately for ex vivo bioluminescence imaging. The excised tumors were fixed with 10% neutral formalin tissue fixative. Prussian blue-eosin double staining was performed on 5 mm paraffin slices by experienced pathologists with blind samples.

### 2.11. In Vivo Photothermal Conversion

SPIOCs@HSA(PTX)-RGD NPs were injected into tumor-bearing rats. Under isofluorane anesthesia, the tumor sites were exposed to 808 nm laser irradiation (0.5 W/cm^2^) 10-hour postinjection. The temperature changes were recorded by an infrared thermal imager.

### 2.12. In Vivo Antitumor Efficiency

U87 glioma subcutaneous xenograft models were randomly divided into 4 groups (*n* = 5), and different treatments were administered by tail vein intravenous injection. The groups were (1) PBS, (2) SPIOCs@HSA(PTX), (3) SPIOCs@HSA(PTX)-RGD, and (4) SPIOCs@HSA(PTX)-RGD+808 nm laser irradiation at 10 h postinjection. The tumor volume (Equation (1)) was recorded every two days with a vernier caliper.

(1) $$\begin{array}{c} \text{Tumor volume }\left(V\right)=\left(\text{major axes}\times \text{minor }{\text{axes}}^{2}\right)\times 0.5, \end{array}$$

(2) $$\begin{array}{c} \text{Relative value }\left(V_{\text{r}}\right)=V/V_{0}\left(V_{0}:\text{original tumor volume}\right). \end{array}$$

Equation (2) was used to calculate the relative tumor volume changes. The treatments began when the tumor size reached approximately 100 mm^3^, and the rats were sacrificed after 14 days. The tumors were dissected for weight measurement and further study.

### 2.13. In Vivo ROS and Apoptosis Detection

To analyze the ROS accumulation, frozen tumor sections from each group at 4-day posttreatment were incubated with the ROS-sensitive probe dihydroethidium (DHE) for 30 min. The nuclei were labeled with DAPI. The fluorescence was detected by fluorescence microscopy.

To detect the apoptosis induced by the ROS burst, a TUNEL apoptotic fluorescence assay was performed. In brief, frozen slices taken at day 4 after different treatments were fixed with 4% formalin, blocked with 3% H_2_O_2_, and immersed in permeable solution. The slices were incubated with the TUNEL solution containing TDT enzyme and fluorescent labeling solution for 1 h in the dark. After washing the slices with PBS three times, the nuclei were labeled with DAPI. Apoptosis was detected via the stains by fluorescence microscopy. The fluorescence staining experiments above were all conducted and analyzed by experienced pathologists with blind samples.

### 2.14. Statistics

GraphPad Prism 7.0 was used for statistical analysis. Student's *t*-test was utilized to assess differences between distinct groups. *P* values less than 0.05 were supposed to be statistically significant. Experimental results were presented as the mean ± standard deviation.

## 3. Results and Discussion

### 3.1. Synthesis and Characterization

A solvothermal synthesis protocol was applied to prepare ultrasmall Fe_3_O_4_ nanoparticles. Hydrophobic ultrasmall superparamagnetic iron oxide nanoclusters (SPIOCs), which were employed as the core of our SPIOCs@HSA(PTX)-RGD nanoplatform, were shelled by the hydrophilic human serum albumin (HSA). The SPIOCs played an important role in chemodynamic therapy and photothermal conversion. HSA was decorated on SPIOCs for long-term blood circulation via a self-assembly method. PTX, which could inhibit mitosis and trigger cancer cell apoptosis, was used as the shell and coated onto the surface of the SPIOCs via hydrophobic interactions with HSA. To increase tumor specificity, RGD peptide was conjugated onto the SPIOCs@HSA(PTX) NPs for targeting the abundant *α*_v_*β*_3_-integrin on the glioma cells.

**Figure 2:**
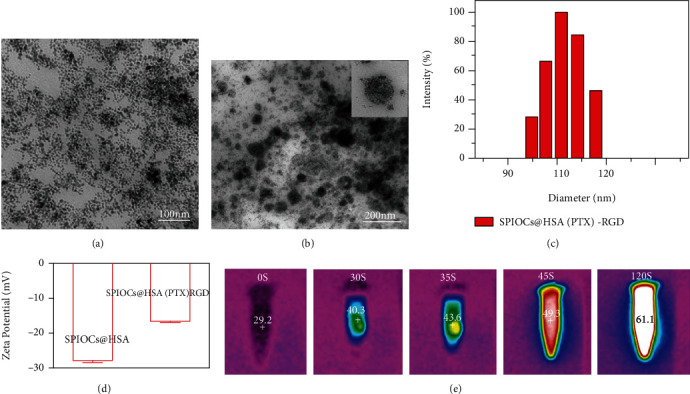
TEM morphologies of (a) ultrasmall superparamagnetic Fe_3_O_4_ NPs and (b) SPIOCs@HSA(PTX)-RGD; (c) Size distribution determined by DLS; (d) zeta potential of SPIOCs@HSA and SPIOCs@HSA(PTX)-RGD NPs; (e) photothermal temperatures of the SPIOCs@HSA(PTX)-RGD irradiated by 808 nm light (0.5 W/cm^2^).

The morphology and structure were identified by transmission electron microscopy (TEM). Images of the ultrasmall superparamagnetic Fe_3_O_4_ NPs (Figure 2(a)) showed a relatively uniform spherical morphology of which the diameter was less than 10 nm. In order to obtain monodispersed SPIOCs@HSA(PTX)-RGD nanoclusters, an ultrasound vibration-assisted self-assembly technique had been applied in our preparation. The morphology of SPIOCs@HSA(PTX)-RGD nanoclusters was revealed by TEM images (Figure 2(b)), which were uniformly dispersed with an average diameter ≈ 95 nm. The typical hydrodynamic radius of the SPIOCs@HSA(PTX)-RGD (112 ± 10.5 nm) was obtained via dynamic light scattering (DLS) (Figure 2(c)). The zeta potential (surface charge) of the SPIOCs@HSA(PTX)-RGD NPs (~-16 mV) was higher than that of the SPIOCs@HSA NPs (~-28 mV) (Figure 2(d)), indicating a more stable structure and less resistance for the internalization in glioma cells.

### 3.2. In Vitro Photothermal Conversion

Near-infrared (NIR) laser light can penetrate biological tissues more efficiently and inflicts less damage than ultraviolet light. The excellent photothermal conversion effect of our SPIOCs@HSA(PTX)-RGD NPs was mainly attributed to the SPIOCs core. When irradiated with 808 nm NIR laser light (0.5 W/cm^2^), the temperature of SPIOCs@HSA(PTX)-RGD NPs solution increased rapidly by nearly 20 degrees during the initial 45 s and continued to increase slowly over the subsequent period (Figure 2(e)). Within 120 s, the temperature increased by more than 30°C and nearly reached 61.1°C, which resulted in the sufficient destruction of tumor cells.

### 3.3. In Vitro Endocytosis and Cytotoxicity

**Figure 3:**
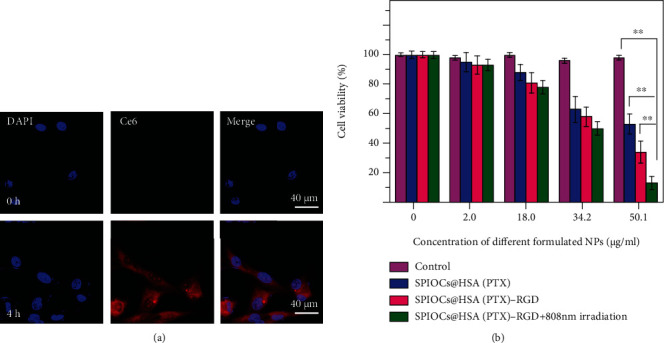
(a) Internalization of Ce6-labeled SPIOCs@HSA-RGD into U87 cells after incubation for 4 h; (b) in vitro antitumor ability of SPIOCs@HSA(PTX)-RGD at different PTX concentrations (^∗∗^*P* < 0.01).

To evaluate the capacity of the SPIOCs@HSA-RGD NPs to target human glioma U87 cells overexpressing *α*_v_*β*_3_ integrin, an uptake study was performed, and the internalization was investigated by confocal microscopy. The SPIOCs@HSA-RGD NPs were labeled with fluorescent Chlorin e6 (Ce6). After incubation with the U87 cells for 4 hours, strong Ce6 red fluorescence signals were observed both in the cytoplasm and nucleus (Figure 3(a)). This endocytosis might be attributed to the HSA and RGD peptide on the particles interacting with the albumin gp60 receptor and *α*_v_*β*_3_ integrins overexpressed on the surface of glioma cells.

Moreover, the biocompatibility and cytotoxicity of this nanoplatform were evaluated by a cell viability assay (Figure 3(b)). SPIOCs@HSA(PTX)-RGD did not show significant cytotoxicity towards U87 cells when the concentration of PTX was below 18 *μ*g/mL. The inhibitory effect of the different NP groups on the U87 cells was evaluated. Interestingly, SPIOCs@HSA(PTX)-RGD was much cytotoxic than those without RGD peptide decoration at a PTX concentration of 34.2 *μ*g/mL; further, the similar phenomenon was observed when the PTX concentration exceeded 50 *μ*g/mL. And this phenomenon was attributed to the RGD targeting capacity to U87 cells, which was beneficial for tumor inhibition. The synergistic SPIOCs@HSA(PTX)-RGD+808 nm irradiated group resulted in the best antitumor effect among all the groups, which demonstrated the superiority of photothermally mediated chemo/chemodynamic therapy.

### 3.4. In Vivo Dynamic Biodistribution

**Figure 4:**
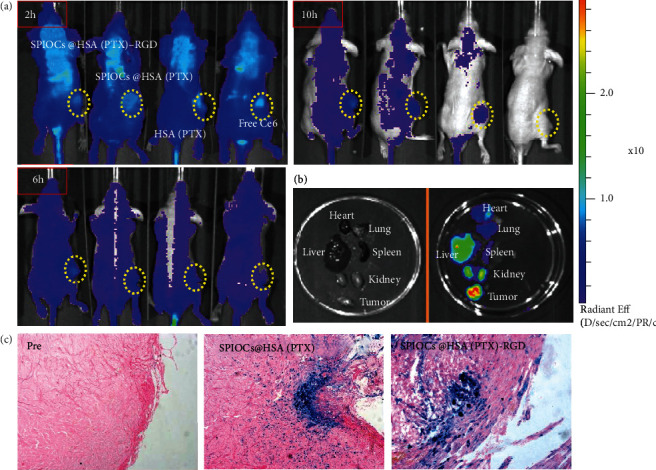
(a) Biodistribution of different nanoparticle groups recorded by IVIS fluorescence images at 2-hour, 6-hour, and 10-hour postinjection. (b) Bioluminescence signals of ex vivo tumor and normal organs including the heart, lung, liver, spleen, and kidneys of SPIOCs@HSA(PTX)-RGD group. (c) Prussian blue-eosin double staining of tumor sections. Prussian blue-stained regions represented the iron nanoparticles deposited in tumor beds 10-hour postinjection.

To investigate the accumulation and distribution of the SPIOCs@HSA(PTX)-RGD NPs in the tumor environment, Ce6-labeled NPs were intravenously injected into U87-bearing mice, and their in vivo biodistribution was evaluated at different time points (Figure 4(a)). Over time, the fluorescence signal intensity at the tumor site increased, whereas it gradually decreased at other sites. Six-hour postinjection, the aggregation of the SPIOCs@HSA(PTX)-RGD in the tumor environment was more pronounced than that of SPIOCs@HSA(PTX), HSA(PTX), and free Ce6 groups, indicating good targeting capability. After 10 hours, free Ce6 was completely biocleared, while a large amount of SPIOCs@HSA(PTX)-RGD NPs were retained in the blood circulation, showing the longest half-value period among the different groups. The fluorescence biodistribution in the tumors and major organs at 10-hour postinjection also confirmed the specific targeting capability of our SPIOCs@HSA(PTX)-RGD NPs (Figure 4(b)). Furthermore, Prussian blue/eosin staining of tumor specimen slices (Figure 4(c)) indicated the iron distribution (blue region) in the tumor tissue 10 hours after injection. The SPIOCs@HSA(PTX)-RGD group showed the maximum density of iron compared with the other groups, indicating that iron oxide nanoparticles penetrated into the interior of the tumor tissues effectively, which was consistent with the fluorescence results.

### 3.5. In Vivo Synergistic Therapy for Glioma

**Figure 5:**
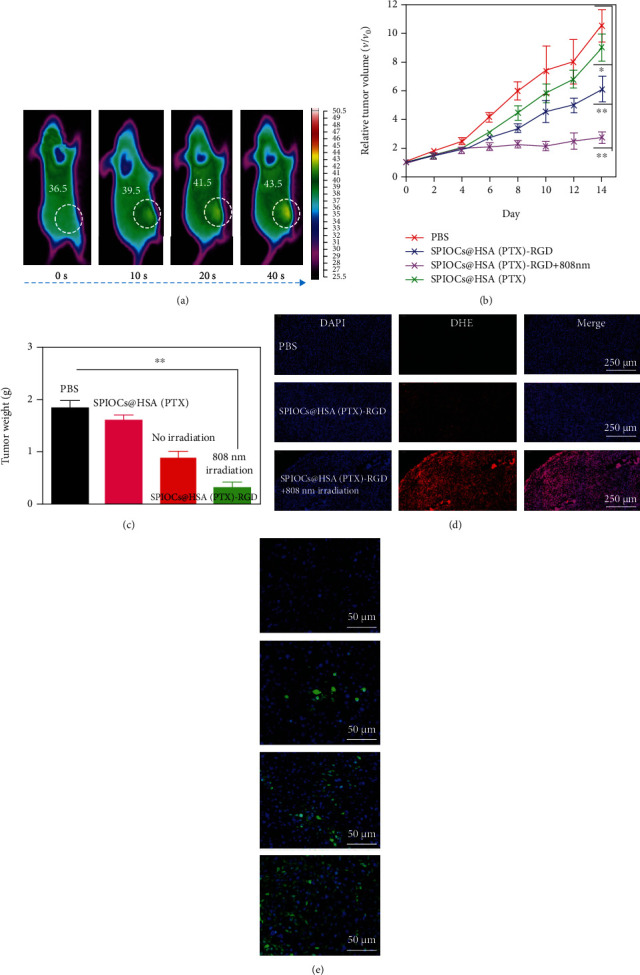
(a) Real-time infrared thermal images of tumor-bearing rats exposed to laser irradiation (808 nm, 0.5 W/cm^2^) 10 hours after the injection of SPIOCs@HSA(PTX)-RGD NPs; (b) in vivo tumor growth curves of the glioma-bearing mice after the different treatments (^∗^*P* < 0.05, ^∗∗^*P* < 0.01); (c) tumor weights measured after the various treatments (^∗∗^*P* < 0.01); (d) oxidative stress detected by ROS probe; (e) TUNEL-stained tumor slices taken at day 4; groups from top to bottom are PBS, SPIOCs@HSA(PTX), SPIOCs/@HSA(PTX)-RGD, and SPIOCs@HSA(PTX)-RGD+808 nm irradiation, respectively.

The photothermal effect of SPIOCs@HSA(PTX)-RGD NPs in vivo was verified by full-body infrared thermography of nude rats bearing U87 glioma. After intravenous injection of the SPIOCs@HSA(PTX)-RGD NPs, hyperthermia at the tumor site was observed in real time when irradiated with NIR light (808 nm, 0.5 W/cm^2^) (Figure 5(a)), indicating an excellent photothermal conversion in vivo.

To further examine the tumor inhibition ability, the corresponding treatments were applied to subcutaneous U87 glioma, and the tumor volumes were recorded to evaluate the respective antitumor effects. The SPIOCs@HSA(PTX)-RGD NP group most efficiently inhibited the tumor growth compared with the other groups treated with free PBS solution and SPIOCs@HSA(PTX) NPs. When synergistic therapy of SPIOCs@HSA(PTX)-RGD NPs + NIR light irradiation was applied, the tumor growth was inhibited even more (Figure 5(b)). Compared with groups without NIR irradiation, a substantial difference was observed in the tumor weights after 14 days (Figure 5(c)).

The production of reactive oxygen species (ROS) and oxidative stress were determined by the ROS probe (DHE). Slices of the tumors treated with synergistic therapy of SPIOCs@HSA(PTX)-RGD NPs + NIR light irradiation were remarkably brighter with a more intense red fluorescence than those without irradiation (Figure 5(d)). This result showed that a large amount of ROS, such as superoxide anions and hydroxyl radicals, were produced by the synergistic therapy. These ROS were produced by the SPIOC-catalyzed Fenton reaction at elevated temperature due to photothermal conversion. The ROS generation mediated by photothermal conversion synergistically enhanced the cell apoptosis, which could be visualized by the apoptosis fluorescence of the tumor slices taken at day 4 (Figure 5(e)). All the results indicated that our synergistic therapy is promising for application in vivo.

## 4. Conclusion

In summary, we developed an efficient SPIOCs@HSA(PTX)-RGD nanoplatform for photothermal-mediated chemo/chemodynamic therapy. Superparamagnetic iron oxide nanoclusters (SPIOCs) triggered photothermal conversion and efficiently catalyzed the Fenton reaction. Coating with human serum albumin (HSA) resulted in high stability and biocompatibility of the nanoplatform. Conjugated RGD peptide ligands allowed for efficient targeting of human U87 glioma. With the assistance of NIR light irradiation, excessive cytotoxic ROS generated from photothermally mediated Fenton reaction coupled with chemotherapy resulted in glioma apoptosis. This combination therapy was more effective in promoting glioma ablation than any single treatment. Considering the glioma growth inhibition, specific targeting ability, and biosafety, our photothermal-mediated synergistic chemo/chemodynamic treatment is promising for use in the clinic.

## Data Availability

The data used to support the findings of this study are included within the article.
